# Halide Perovskite: A Rich Source of Thermal Insulator

**DOI:** 10.1002/advs.76333

**Published:** 2026-06-26

**Authors:** Haolin Ye, Bangzhi Ge, Yuan Yu, Chongjian Zhou

**Affiliations:** ^1^ State Key Laboratory of Solidification Processing School of Materials Science and Engineering Northwestern Polytechnical University Xi'an China; ^2^ Institute of Physics (IA) RWTH Aachen University Aachen Germany

**Keywords:** anharmonicity, condensed matter physics, halide, heat capacity, materials science, phonon, phonon scattering, thermal conduction, thermal conductivity, thermal transport

## Abstract

Low lattice thermal conductivity is a key physical parameter for realizing efficient thermal management and energy conversion. Halide perovskites have emerged as an ideal platform for exploring the physics of extreme thermal transport and for designing novel thermal management materials, owing to their rich structural tunability and intrinsically ultralow thermal conductivity. This review discusses the origins of ultralow thermal conductivity in halide perovskites, spanning from macroscopic thermal phenomena to microscopic phonon transport. Halide perovskites exhibit characteristic thermal signatures, including weak temperature dependence of thermal conductivity, a boson‐like peak in heat capacity, and low sound velocities. These properties stem from a soft crystal lattice associated with metavalent bonding, as well as from strong anharmonic phonon scattering induced by lattice disorder and rattling modes. Such glass‐like lattice dynamics lead to an anomalous accumulation of low‐frequency phonons and intense phonon scattering, pushing phonons toward the Ioffe‐Regel limit and causing a breakdown of the conventional phonon gas model. Therefore, the inclusion of coherent phonons is essential for properly describing the intrinsic “phonon glass” character of these materials.

## Graphical abstract

Halide perovskites exhibit ultralow thermal conductivity driven by intrinsic lattice softness and strong anharmonicity, falling below conventional defect‐engineering limits. Weak metavalent bonding, A‐site rattling, and dynamic octahedral tilting drive phonon scattering to the Ioffe–Regel limit, where wave‐like tunneling replaces particle‐like propagation. This phonon‐glass behavior establishes halide perovskites as a universal platform for thermal insulation design.

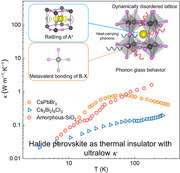

## 1 Introduction

Energy has emerged as a cornerstone underpinning high‐tech industries in the 21^st^ century, ranging from advanced chips to intelligent robotics. During the utilization and conversion of high‐grade energy, approximately two‐thirds of which is inevitably dissipated as low‐grade heat [1, 2, 3]. For instance, conventional thermal power generation operates at an efficiency of merely ∼35%, with the majority of energy lost to the surroundings [4]. Consequently, intensive efforts are being directed toward two principal technological pathways: waste heat recovery (thermoelectric conversion) and thermal suppression (thermal insulation materials), aiming to reclaim energy losses on the scale of several terawatt‐hours annually [5, 6, 7, 8]. Central to these energy technologies is the rational design of materials with intrinsically low thermal conductivity, which can serve as effective thermal barriers to enable precise control over heat transfer and conversion.

Within the framework of phonon gas kinetic theory, the lattice thermal conductivity is expressed as κ = 1/3C_v_ν*l*, where C_v_, *ν*, and *l* denote the volumetric heat capacity, the phonon group velocity, and the phonon mean free path (MFP), respectively [9]. The *ν* is dictated by the phonon dispersion arising from the crystal structure, whereas *l* is governed by phonon scattering. The reduction of the MFP and the attendant suppression of thermal conductivity constitute the central objectives of conventional defect engineering strategies, which rely on the introduction of extrinsic phonon scattering centers such as point defects and dislocations [10, 11]. Nevertheless, in crystalline materials, the MFP is fundamentally bounded below by half the phonon wavelength or the average interatomic spacing, thereby establishing a physical limit to the extent to which extrinsic scattering can modulate thermal conductivity [12]. As a result, the focus of research has progressively shifted toward exploring material systems that possess intrinsically strong phonon scattering and inherently low sound velocities [13]. By elucidating the unique thermal transport mechanisms derived from their distinctive crystal structures, the ultimate goal is to establish systematic design principles that correlate low thermal conductivity with specific microstructural features.

**FIGURE 1:**
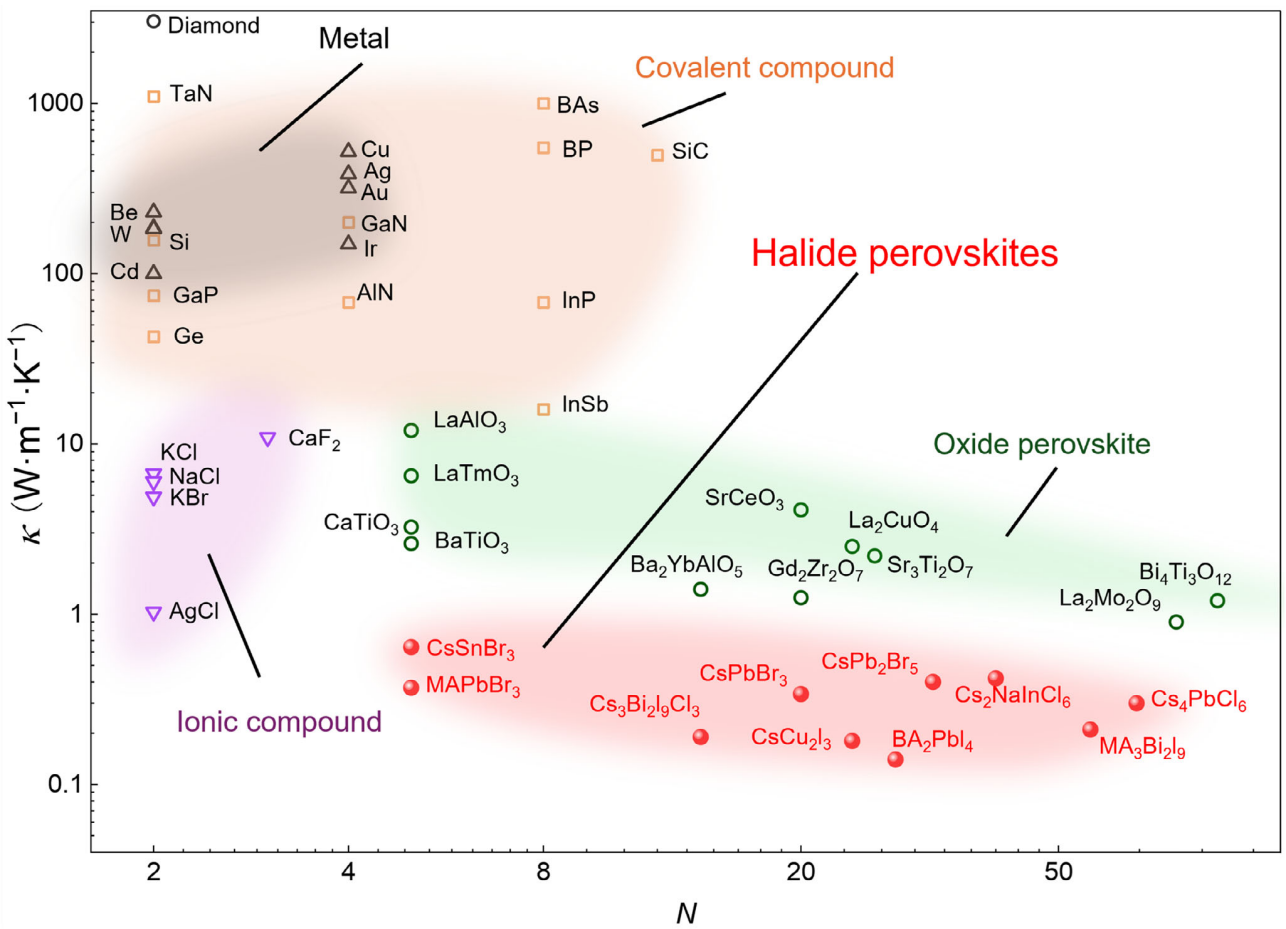
Thermal conductivity and the number of atoms per unit cell ***N*** of various materials (metal [18, 24]; covalent compound [16, 17, 25, 26, 27, 28, 29, 30, 31, 32, 33, 34, 35]; ionic compound [15, 36]; oxide perovskite [37, 38, 39, 40, 41, 42, 43, 44, 45]), in comparison with halide perovskites [46, 47, 48, 49, 50, 51, 52, 53, 54, 55, 56].

According to lattice dynamics theory, as the number of atoms per unit cell (*N*) increases, the total number of phonon branches (3*N*) rises accordingly, with a marked proliferation of optical branches (3*N*‐3). This substantial enrichment of optical phonon modes greatly expands the phase space for coupling and scattering channels between acoustic and optical phonons, leading to a precipitous decline in lattice thermal conductivity [14]. Consequently, a general empirical rule emerges: the thermal conductivity of a crystal tends to decrease with an increasing *N*, as illustrated in Figure 1. Concurrently, the nature of chemical bonding governs both the rigidity of the crystal lattice and the localization state of electrons, thereby dictating disparities in sound velocity and electronic thermal conduction. As a result, the thermal conductivities of ionic compounds (KCl‐6.68 W·m^−^^1^·K^−^^1^, NaCl‐5.89 W·m^−^^1^·K^−^^1^) [15] are substantially lower than those of strongly covalent compounds (Si‐157 W·m^−^^1^·K^−^^1^ or TaN‐1100 W·m^−^^1^·K^−^^1^) [16, 17] or metals possessing abundant free electrons (Au‐385.7 W·m^−^^1^·K^−^^1^, Ag‐318 W·m^−^^1^·K^−^^1^) [18], as presented in Figure 1.

The “large unit cell” design, in conjunction with “chemical bond engineering”, thus constitutes a fundamental strategy for realizing materials with intrinsically low lattice thermal conductivity [9]. The synergy of these two approaches has led to the emergence of numerous low *κ* materials exhibiting distinctive lattice dynamics, as extensively documented in recent literature. Representative examples include the rattling vibration of guest atoms in cage‐like compounds such as Ba_8_Ga_16_Ge_30_ [19]; the intense phonon scattering induced by liquid‐like ion migration in superionic conductors such as Ag_8_SnS_6_ [20] and Cu_2_Se [21]; and the pronounced lattice anharmonicity arising from inhomogeneous chemical bonding in simple binary compounds, e.g., SnSe [22] and InTe [23]. However, these manifestations of ultralow *κ* are typically contingent upon specific structural motifs or chemical compositions, rendering the underlying design strategies difficult to systematize or transplant to other material classes. Consequently, it remains challenging to establish a material platform that simultaneously offers both tunability and universality for low *κ*.

Halide perovskites, as an emerging class of semiconductor materials, exhibit a rich compositional and structural landscape coupled with remarkably convergent ultralow thermal conductivity, as illustrated in Figure 1, thereby positioning them as ideal templates for the design of thermal insulation materials [46, 47, 48, 49, 50, 51, 52, 53, 54, 55, 56, 57, 58]. They adopt the general structural formula ABX_3_: the A‐site accommodates monovalent cations (Cs^+^, MA^+^), the B‐site hosts various metal ions (Pb^2+^, Bi^3+^, Ag^+^), and the X‐site is occupied by halide ions (F^−^, Cl^−^, Br^−^, or I^−^). Through compositional variation, halide perovskites manifest a wide spectrum of geometric configurations and symmetries [53, 54, 59, 60]. For instance, three‐dimensional (3D) CsPbBr_3_, two‐dimensional (2D) CsPb_2_Br_5_, and zero‐dimensional (0D) Cs_4_PbBr_6_ are distinguished by the connectivity modes of the [PbBr_6_] octahedra. Notably, as shown in Figure 1, oxide perovskites share analogous crystal structures and similar tunability advantages with halide perovskites. However, their *κ* values are approximately an order of magnitude higher, as exemplified by the contrast between CsSnBr_3_ (0.6 W·m^−^^1^·K^−^^1^) [46] and CaTiO_3_ (4 W·m^−^^1^·K^−^^1^) [39]. This striking divergence in thermal transport properties between structurally homologous systems constitutes a highly compelling anomaly in lattice heat conduction. Therefore, elucidating the unique mechanisms underpinning the ultralow thermal conductivity shared by structurally diverse halide perovskites holds promise for unraveling their “phonon glass” thermal transport characteristics. Moreover, it also provides a universal design pathway for realizing high‐performance thermal insulation materials through intrinsic phonon engineering [61].

This review examines the ultralow thermal conductivity of halide perovskites and its origins in chemical bonding and lattice dynamics, with a focus on ABX_3_‐structured halide perovskites along with several other halide perovskites of special structural types (e.g., Cs_3_Bi_2_I_6_Cl_3_, Cs_3_Bi_2_I_9_, Cs_2_PbI_2_Cl_2_, etc.). We first discuss the macroscopic signatures of low *κ* in halide perovskites. The weak temperature dependence, the emergence of boson‐like peaks in low‐temperature heat capacity, and the low sound velocities collectively reflect their intrinsically soft lattices and pronounced anharmonicity. We then survey recent advances in understanding the low *κ* of halide perovskites and summarize the common physical mechanisms underlying their structural, bonding, and lattice dynamic characteristics. Finally, we delve into the microscopic phonon transport picture to elucidate the “phonon glass” thermal transport behavior exhibited by halide perovskites. Through this comprehensive overview and critical assessment of recent research on the ultralow *κ* of halide perovskites, we aim to provide insightful material perspectives. We also establish design guidelines for exploring the fundamental limits of solid‐state heat conduction and for developing materials with tailored anisotropic thermal transport properties.

## 2 The Classic Ultralow κ Characteristic of Halide Perovskites

The potential of halide perovskite materials in realizing ultralow thermal conductivity is attracting increasing attention. For instance, halide perovskite Cs_2_HgPtCl_6_ has been reported to exhibit a room‐temperature thermal conductivity approaching that of air [71]. Such anomalously low thermal conductivity manifests several distinctive features in macroscopic physical property measurements. First, the *κ* in the high‐temperature regime follows a dependence of *κ* ∝*T* ^−^^n^ with n<1, which deviates markedly from the *T* ^−^^1^ decay characteristic of conventional crystals [49]. Second, low‐temperature specific heat measurements reveal a boson‐like peak in the *C*_p_/*T* ^3^ curve [46]. Third, these materials display ultralow sound velocities approaching those of liquids. These macroscopic signatures not only furnish quantitative metrics for assessing and benchmarking the ultralow *κ* of halide perovskites but also collectively point to a common origin rooted in an exceptionally soft lattice framework and strong dynamic anharmonicity.

**FIGURE 2:**
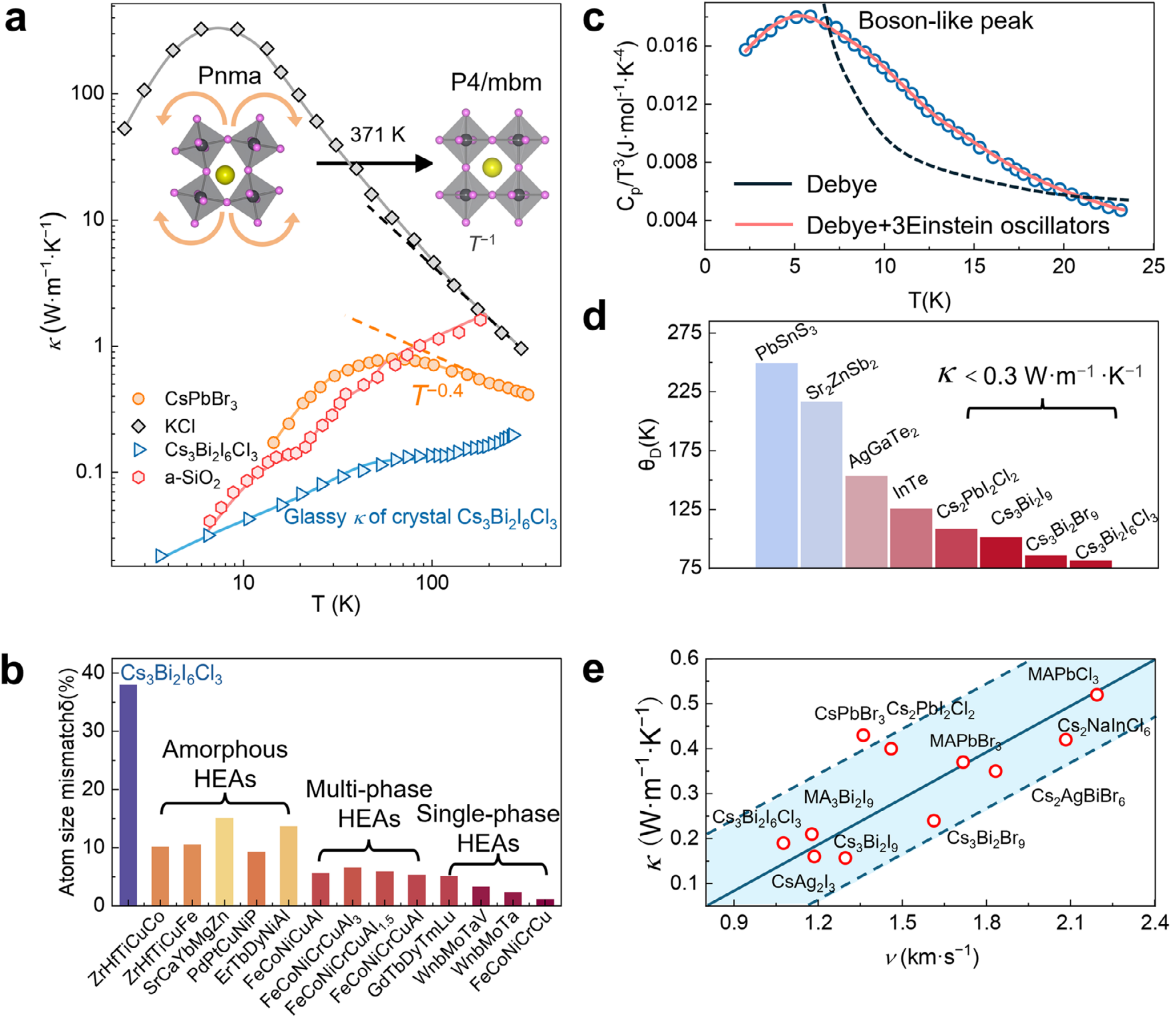
Thermal characteristics of halide perovskites. (a) The temperature‐dependent thermal conductivity for KCl [62], amorphous SiO_2_ [63], CsPbBr_3_ [55], and Cs_3_Bi_2_I_6_Cl_3_ [63] below 300 K: Classical T^−^^1^ dependence for KCl, T^−^^0.4^ for CsPbBr_3_, and glassy behavior for Cs_3_Bi_2_I_6_Cl_3_ and a‐SiO_2_. Inset: schematic illustration of octahedral rotations during the phase transition, with orthorhombic and tetragonal CsPbBr_3_ as examples. (b) The lattice distortion of Cs_3_Bi_2_I_6_Cl_3_ compared with traditional high‐entropy alloys. Reproduced with permission [63]. Copyright 2025, National Academy of Sciences. (c) Temperature dependence of C_p_/T^3^ for Cs_2_PbI_2_Cl_2_ showing a boson‐like peak that deviates from the Debye model (black dashed line), fitted using the Debye‐Einstein model (red line) [64]. (d) Low Debye temperature (*θ*_D_) of halide perovskites [47, 64, 65, 66] compared with other materials [11, 23, 67, 68]. (e) Low *κ* correlates with low sound velocity (*ν*) in halide perovskites [47, 49, 50, 64, 65, 69, 70].

Thermal conductivity in crystals is conventionally governed by boundary scattering and phonon‐phonon Umklapp scattering. It follows the classical relationship of *κ* ∝*T* ^3^ at low temperatures and *κ* ∝ *T* ^−^^1^ above the Debye temperature (*θ*_D_). However, the temperature exponent of *κ* in halide perovskites is typically greater than −1. For instance, as shown in Figure 2a, KCl exhibits *T* ^−^^1^ near 300K, whereas CsPbBr_3_ displays *T* ^−^^0.4^ [55, 62]. This behavior stems from the exceptionally high sensitivity of the perovskite lattice to temperature under the combined influence of third‐ and fourth‐order anharmonicity [49]. Taking CsPbBr_3_ as an example, it undergoes successive phase transitions from orthorhombic to tetragonal at 371 K and then to cubic at 403 K upon heating [59]. These transitions are accompanied by rotations of the [PbBr_6_] octahedra as illustrated in the inset of Figure 2a. Even within a single‐phase region, the [PbBr_6_] octahedra continue to tilt and oscillate with changing temperature [72]. Consequently, the phonon dispersion relations determined by the crystal structure are not static but undergo real‐time dynamic renormalization with temperature. Phonon frequencies and group velocities evolve continuously, and the structure of the scattering phase space is accordingly modified [73]. This strong anharmonicity induces phonon dispersion renormalization and renders the conventional three‐phonon scattering model invalid. If the temperature evolution of the interatomic force constants is artificially frozen in calculations of perovskite thermal conductivity, the predicted thermal conductivity reverts to the classical *T* ^−^^1^ [74].

Notably, Cs_3_Bi_2_I_6_Cl_3_ within the halide perovskite family exhibits a rare glass‐like thermal transport behavior while retaining the crystalline features of a long‐range ordered lattice [47]. As shown in Figure 2a, the *κ* of Cs_3_Bi_2_I_6_Cl_3_ increases with temperature at low temperatures yet lacks the characteristic Umklapp peak of a crystalline material and gradually approaches the glass limit at elevated temperatures. This behavior closely resembles the thermal transport process of amorphous SiO_2_. Such anomalous heat conduction originates primarily from its highly distorted structure [63]. The Cs_3_Bi_2_I_6_Cl_3_ crystal derives from Cs_3_Bi_2_I_9_ through the substitution of I^−^ with Cl^−^, which bridges two Bi^3+^ centers within the 0D [Bi_2_I_9_] cluster [60]. The substantial difference in atomic radius between Cl and the other constituent atoms induces severe lattice distortion in the Cs_3_Bi_2_I_6_Cl_3_ lattice. As depicted in Figure 2b, this pronounced lattice distortion approaches 40% and is even more significant than that observed in conventional high‐entropy alloys (HEAs) [63]. Consequently, the atomic positions freeze at low temperatures at locations that deviate from the ideal lattice sites. At room temperature, they readily surmount a tiny energy barrier of merely 0.7 meV/atom to hop among nearby equilibrium positions [63]. This dynamic disorder closely mimics the disordered state of amorphous glasses and scatters phonons profoundly.

The glass‐like and weak temperature dependence of *κ* in halide perovskites reflects a profound suppression of their lattice vibrational modes. Heat capacity (*C*_p_)serves as a probe capable of revealing the energy absorption capacity of lattice vibrational modes (phonons) and capturing their vibrational signatures [75]. At low temperatures, the vibrational modes of high‐frequency phonons are frozen out, and only low‐frequency phonons are excited. Consequently, the heat capacity drops sharply with decreasing temperature following the Debye law as *T* ^3^ [76]. However, the *C*_p_/*T* ^3^ vs. T plot of halide perovskites exhibits an anomalous boson‐like peak below 30 K that deviates from the Debye model, as shown in Figure 2c. Such a feature is commonly observed in heavily defective or amorphous materials [77]. These modes undergo resonant scattering with transverse acoustic branches of similar frequencies, thereby blocking the efficient propagation of heat‐carrying phonons [78]. In experimental analyses, the contributions of these additional localized vibrations are typically quantified and fitted using a combined Debye‐Einstein model. The corresponding expression can be written as [79]

$$\frac{C_{P}}{T}=\gamma +\beta T^{2}+\sum A_{n}\left(\theta_{E_{n}}^{2}\right){\left(T^{2}\right)}^{-\frac{3}{2}}\frac{e^{\frac{\theta_{E_{n}}}{T}}}{{\left(e^{\frac{\theta_{E_{n}}}{T}}-1\right)}^{2}}$$

where γ and β*T* ^2^ represent the electronic and Debye lattice contribution to the heat capacity, respectively, and the final term represents the contribution from localized Einstein oscillators. Raman spectroscopy and first‐principles density functional theory (DFT) calculations have confirmed that these localized modes originate predominantly from a multitude of low‐frequency vibrational modes involving the A‐site cations and halogen atoms of the perovskite lattice [65, 66]. A full description of the broadening and intensity of the low‐temperature boson‐like peak in halide perovskite systems, therefore, typically necessitates the inclusion of three independent Einstein oscillators. For each Einstein oscillator, its Einstein temperature (*θ*_En_) is related to the characteristic frequency (*ω*_n_) as $\theta_{E_{n}}=\hbar \omega_{n}/k_{B}$. For instance, the fitting of the low‐temperature heat capacity of Cs_2_PbI_2_Cl_2_ in Figure 2c has identified low‐frequency localized modes at 13 cm^−^^1^ (19 K), 31 cm^−^^1^ (45 K), and 61 cm^−^^1^ (88 K) [64]. The group velocities of these phonon branches approach zero, which flattens the phonon dispersion and ultimately yields a thermal conductivity as low as 0.3 W m^−^^1^·K^−^^1^ [64].

Beyond the excess of low‐frequency vibrational states, the fitting parameters derived from heat capacity measurements also reflect the soft lattice characteristics of halide perovskites. The *θ*_D_ can be extracted by fitting low‐temperature heat capacity data and subsequently used to estimate the *ν* of the solid via established relations (θ_D_ = h/k_B_(3*N*/4π*V*)^1/3^ν), thereby providing a quantitative measure of lattice softening [65]. Experimentally, the *ν* of halide perovskites can be directly determined by techniques such as ultrasonic pulse‐echo methods, nanoindentation, or inelastic neutron and X‐ray scattering [65, 70]. A Pearson correlation test reveals a significant negative correlation exceeding −0.4 between the *θ*_D_ or *ν* and the lattice thermal conductivity [69]. These parameters serve as effective descriptors for evaluating and predicting the thermal transport capacity of perovskites. As shown in Figure 2d, the *θ*_D_ of halide perovskites generally falls below 125 K, a value substantially lower than those of conventional semiconductors such as Si (640 K) and other established low‐thermal‐conductivity materials such as InTe (125.89 K) [23, 69]. Correspondingly, as illustrated in Figure 2e, the *ν* values across a wide range of halide perovskite compositions are universally below 2200 m·s^−^^1^ and approach levels typical of water (1500 m·s^−^^1^) [80]. The associated lattice thermal conductivities reside predominantly in the ultralow regime below 0.5 W m^−^^1^·K^−^^1^. The low *ν* originates primarily from the heavy constituent atoms and weak chemical bonding, which in turn are rooted in the distinctive electronic bonding configuration.

## 3 Chemical Bonding and Soft Lattices in Halide Perovskites

Experimental results concerning sound velocity and Debye temperature collectively point to the intrinsically soft lattice character of halide perovskites. We analyze their crystal structure by dividing it into two components to discuss the formation of the soft lattice and the respective contributions to thermal conductivity. One component is the A‐site cation, and the other is the [BX] anionic cluster. The lattice framework is typically constructed from corner‐sharing [BX_6_] octahedra. The A cations occupy the interstitial voids within the octahedral framework through weak electrostatic interactions to neutralize charge and stabilize the structure. The weak bonding combined with the ample space within the octahedral cage affords the A atoms sufficient room to vibrate [61].

**FIGURE 3:**
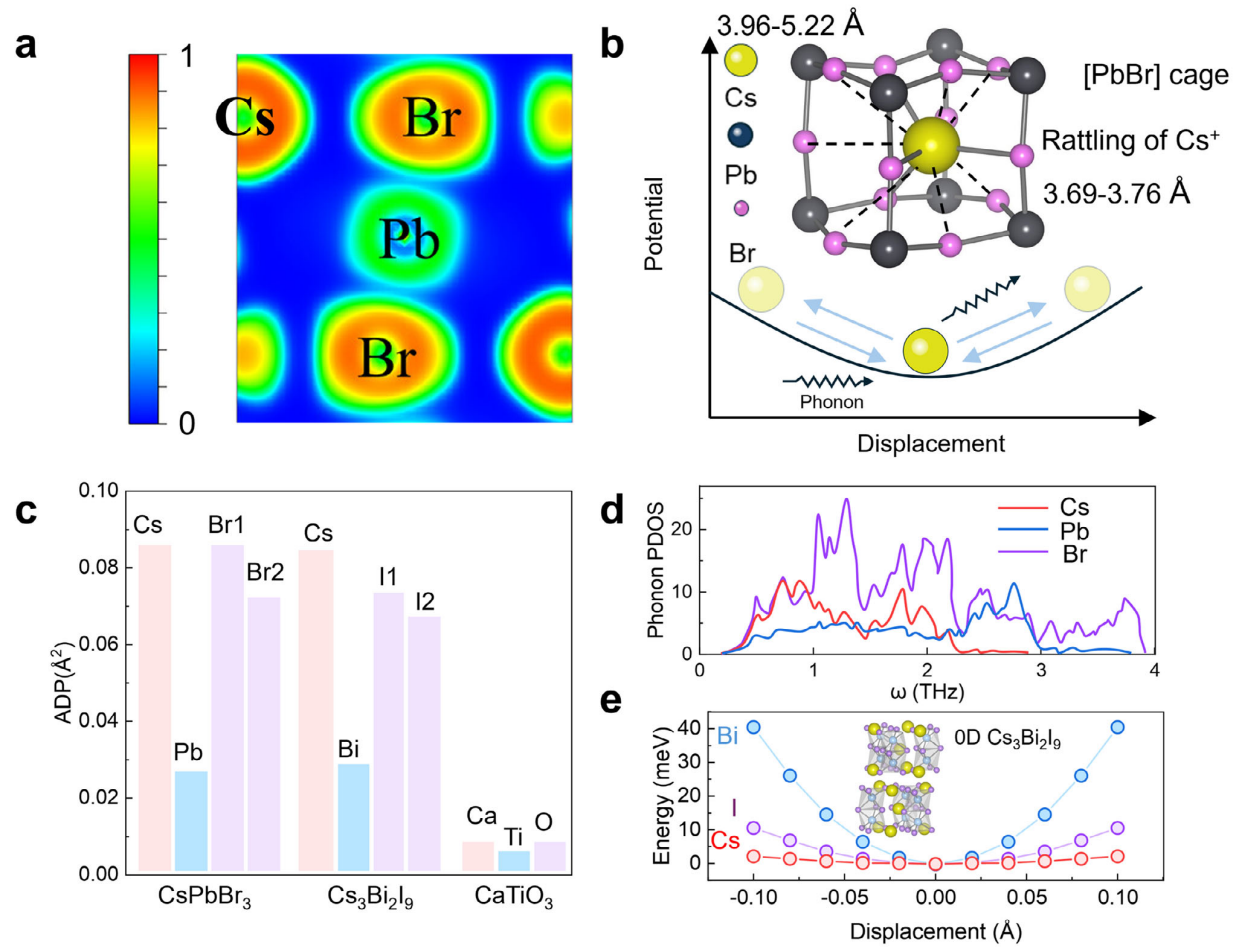
A‐site atom rattling in halide perovskites. (a) Electron localization function (ELF) map of CsPbBr_3_. Reproduced with permission [81]. Copyright 2023, American Chemical Society. (b) Schematic illustration of phonon scattering by A‐site atoms residing on a flat potential energy surface. Inset: A Cs atom situated within the distorted cage formed by [PbBr] polyhedra. (c) Comparison of atomic displacement parameters (ADP) for CsPbBr_3_ [72], Cs_3_Bi_2_I_9_ [65], and CaTiO_3_ [82]. (d) Atom‐resolved contributions to the phonon density of states (PDOS) in CsPbBr_3_ [55]. (e) Atomic displacement potential energy surface of Cs_3_Bi_2_I_9_ [65].

Taking CsPbBr_3_ as an example, its outstanding optoelectronic performance has motivated extensive investigations into its electronic structure and bonding characteristics [81, 83, 84]. The 6s orbitals of Cs atoms contribute negligibly to the band structure and play an almost imperceptible role in the electronic structure [81]. As shown in the electron localization function (ELF) map of Figure 3a, the electrons around Br atoms are delocalized in the 4p orbitals and exhibit clear wavefunction overlap with the 6s/6p orbitals of Pb. In stark contrast, Cs atoms reside in completely isolated regions of space and show no wavefunction overlaps between neighboring atoms.

Furthermore, the inset of Figure 3b reveals that the distances between a Cs atom and its four nearest neighboring Br atoms range from 3.69 to 3.76 Å, values that approach the sum of their ionic radii (r_Cs+_ = 1.7 Å, r_Br−_ = 1.9 Å). The remaining Br atoms are situated at substantially larger distances from the Cs atom, approximately 3.96 to 5.22 Å. This geometry indicates that the Cs atom occupies an off‐center position within a distorted [PbBr] cage and interacts appreciably with only four Br neighbors through predominantly ionic bonding. Under such a low‐symmetry and weakly bonded coordination environment, the Cs atom exhibits exceptionally strong anharmonicity [55]. Experimental structural observations corroborated by extensive computational results demonstrate that Cs atoms reside in pronounced off‐center potential wells within the distorted lattice [85, 86, 87]. The large interstitial void space and weak bonding constraints further give rise to a remarkably flat potential energy surface [85].

In summary, the Pb─Br framework forms an exceptionally loose cage‐like network around the Cs atom and permits the Cs atom to function as a vibrational damper, as illustrated in Figure 3b. This behavior induces intense scattering of phonons, particularly those responsible for thermal transport. In marked contrast, the rattling of A‐site atoms in oxide perovskites is far weaker. As shown in Figure 3c, a comparison of the atomic displacement parameters (ADP) of CsPbBr_3_ and CaTiO_3_ reveals that the atomic thermal vibration in the halide perovskite approaches 0.1 Å^2^, whereas the corresponding value in the oxide perovskite does not exceed 0.01 Å^2^ [72, 82]. From a bonding perspective, the Ca^+^ engages in considerably stronger ionic interactions with its surrounding O^2^^−^ atoms. The ionic radii are r_Ca+_ = 1.1 Å, r_O2−_ = 1.4 Å. Eight Ca─O bonds measure approximately 2.5 Å, and the remaining four bonds exceed the sum of the ionic radii by only 0.7 Å.

The larger ADP value of the Cs atom further substantiates its nearly free state within the lattice. The Lindemann parameter is defined as ξ = APD^1/2^/R_NN_, where R_NN_ denotes the nearest‐neighbor distance [88]. For Cs in CsPbBr_3,_ this parameter is calculated to be 0.076, a value that surpasses the Lindemann melting criterion of 0.07 and thus qualifies the Cs atom as “molten” within the crystalline framework [88]. As anticipated from the low‐temperature heat capacity findings, these loosely bound Cs atoms contribute a substantial density of low‐frequency localized phonon states to the projected phonon density of states (PDOS) of CsPbBr_3_ and give rise to pronounced heavy‐atom rattling scattering, as depicted in Figure 3d [55].

Notably, even when the B─X framework does not form a well‐defined cage, the A‐site atom can still generate scattering effects through localized vibrations. For instance, in Cs_3_Bi_2_I_9_ shown in Figure 3c,e, the [Bi_2_I_9_] clusters exist as zero‐dimensional entities within the lattice, yet the Cs atoms still exhibit remarkably high ADP values and flat potential energy surfaces [65]. This phenomenon is also observed in materials such as Cs_2_SnI_6_ [89], Cs_3_Bi_2_I_6_Cl_3_ [47], and δ‐CsPbI_3_ [55]. These low‐dimensional perovskites can be understood as systems wherein the cage is either unconstrained along certain directions or enlarged, conditions that are both conducive to the free movement of the A‐site atom.

**FIGURE 4:**
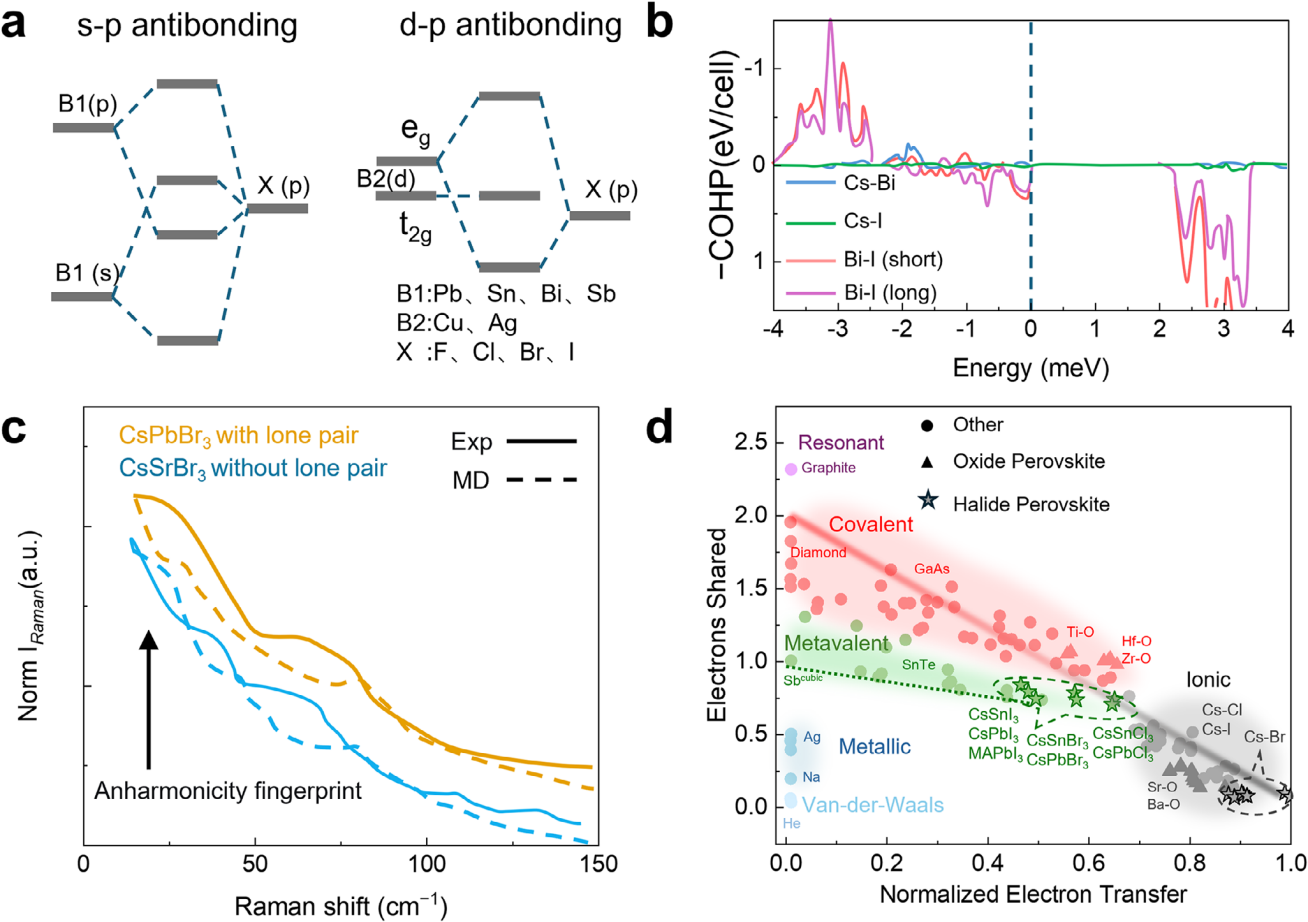
Chemical bonds underpinning lattice softening. (a) Schematic diagram of the antibonding states between B‑site cations and X‑site halogens in halide perovskites. (b) Crystal orbital Hamilton population (COHP) for Cs_3_Bi_2_I_9_. Reproduced with permission [65]. Copyright 2023, Wiley‐VCH GmbH. (c) Comparison of Raman spectra for CsPbBr_3_ (orange) and CsSrBr_3_ (blue) obtained from experiments (solid lines) and molecular dynamics calculations (dashed lines). Reproduced with permission [90]. Copyright 2024, Nature. (d) Two‐dimensional map depicting electron transfer renormalized by oxidation state vs. electron sharing between adjacent atoms for chemical bonding in solids. Reproduced with permission [91]. Copyright 2021, Wiley‐VCH GmbH.

It should be noted that the lattice framework constituted by B─X is inherently soft, as reflected by the weak electron cloud overlap between Pb and Br in Figure 3a, the elevated atomic displacement parameters of the halogen atoms in Figure 3c, and the flat potential energy surface of the I atom approaching the Cs in Figure 3e. This softness directly diminishes the efficiency of atomic thermal vibration transfer through chemical bonds. Numerous studies have proposed that antibonding interactions between B and X weaken the interatomic bonding strength and give rise to weak B─X bonds [81, 92]. Two principal forms of antibonding occur in halide perovskites, as schematically illustrated in Figure 4a. For cations such as Pb^2+^, Sn^2+^ and Bi^3+^, their n‐s^2^ lone‐pair electrons possess relatively high energy and exhibit stereochemical activity [93]. These electrons hybridize with the p orbitals of the halide anions X^−^ to form s‐p antibonding states near the valence band maximum. In contrast, the 3d orbitals of ions such as Cu^+^ and Ag^+^ split into t_2g_ and e_g_ levels under a tetrahedral crystal field. The higher‐lying e_g_ orbitals interact with the p orbitals of X^−^ to form bonding and d‐p antibonding states below the Fermi level, whereas the t_2g_ orbitals remain essentially nonbonding [92]. Experiments reveal that Cs_3_Bi_2_I_9_ exhibits a thermal conductivity of merely 0.2 W m^−^^1^·K^−^^1^ over a broad temperature range from 30 to 50 K [65]. The calculated crystal orbital Hamilton population (COHP) presented in Figure 4b shows that the Bi 6s lone‐pair electrons hybridize with the I 5p orbitals to form extended s‐p antibonding states as evidenced by the negative COHP region below the Fermi level. The Cs orbitals contribute negligibly to bonding or antibonding near the Fermi level. The Bi─I antibonding interactions pervade the lattice framework constructed from [Bi_2_I_9_] clusters and endow the lattice with soft elasticity and pronounced anharmonicity [65].

Researchers have long considered the n‐s2 lone pair electrons of the B‐site cation and the resulting pseudo–Jahn–Teller (PJT) effect to be the primary origin of strong lattice anharmonicity in halide perovskites [94, 95, 96]. A recent study has elegantly challenged this viewpoint through a comparative investigation of CsPbBr_3_ (Pb with 6s^2^ lone pair) and CsSrBr_3_ (Sr with [Kr]5s^0^ and without lone pair) [90]. The two compounds adopt nearly identical crystal structures and undergo the same sequence of phase transitions, yet their electronic structures differ completely [90]. Strikingly, as shown in Figure 4c, both compounds exhibit a pronounced central peak in high‐temperature Raman spectra and molecular dynamics simulations. This central peak serves as a definitive fingerprint of strong anharmonicity [90]. The observation indicates that the lone pair electron effect alone cannot account for the origin of the soft lattice and pronounced anharmonicity in these materials.

We therefore attempt to advance an alternative perspective to elucidate the origin of the soft lattice and strong anharmonicity in halide perovskites. Wuttig et al. have proposed that the exceptional properties of halide perovskites stem from a distinctive metavalent bonding (MVB) character of the B─X bond rather than from lone‐pair effects [91]. MVB represents a unique bond type that lies intermediate among but is distinctive from covalent, ionic, and metallic bonding. It arises from the formation of half‐filled σ‐bonds (2c−1e) between adjacent p orbitals and is accompanied by a finite degree of charge transfer [91]. A recent work indicates that MVB can also be mediated by s‐orbitals, given that the 2c−1e nature is preserved [97]. This bonding scenario can be precisely located and quantified within a 2D map of electron transfer (ET) vs. electron sharing (ES) [98]. As shown in Figure 4d, halide perovskites such as CsSnBr_3_ and CsSnI_3_ reside in the green MVB region. They exhibit ES values close to unity and ET values ranging from 0.4 to 0.6, placing them in the vicinity of materials with pronounced lattice anharmonicity, such as SnTe [97]. In contrast, the Ti─O bonds in oxide perovskites exhibit greater electron sharing and consequently fall within the red covalent bonding region of Figure 4d. The one‐electron bond characteristic of metavalent bonding in halide perovskites is inherently weaker than conventional two‐electron covalent bonds [97, 99]. The B─X bonds in halide perovskites are therefore softer and more susceptible to stretching and bending deformations. In other words, the halogen atoms are themselves weakly bound within the lattice. They undergo large‐amplitude localized vibrations about their lattice sites, thereby softening the phonon modes and giving rise to low Grüneisen parameters and pronounced lattice anharmonicity [99, 100]. This picture also provides a natural explanation for the substantially higher thermal conductivity observed in oxide perovskites.

## 4 Lattice Disorder in Halide Perovskites

**FIGURE 5:**
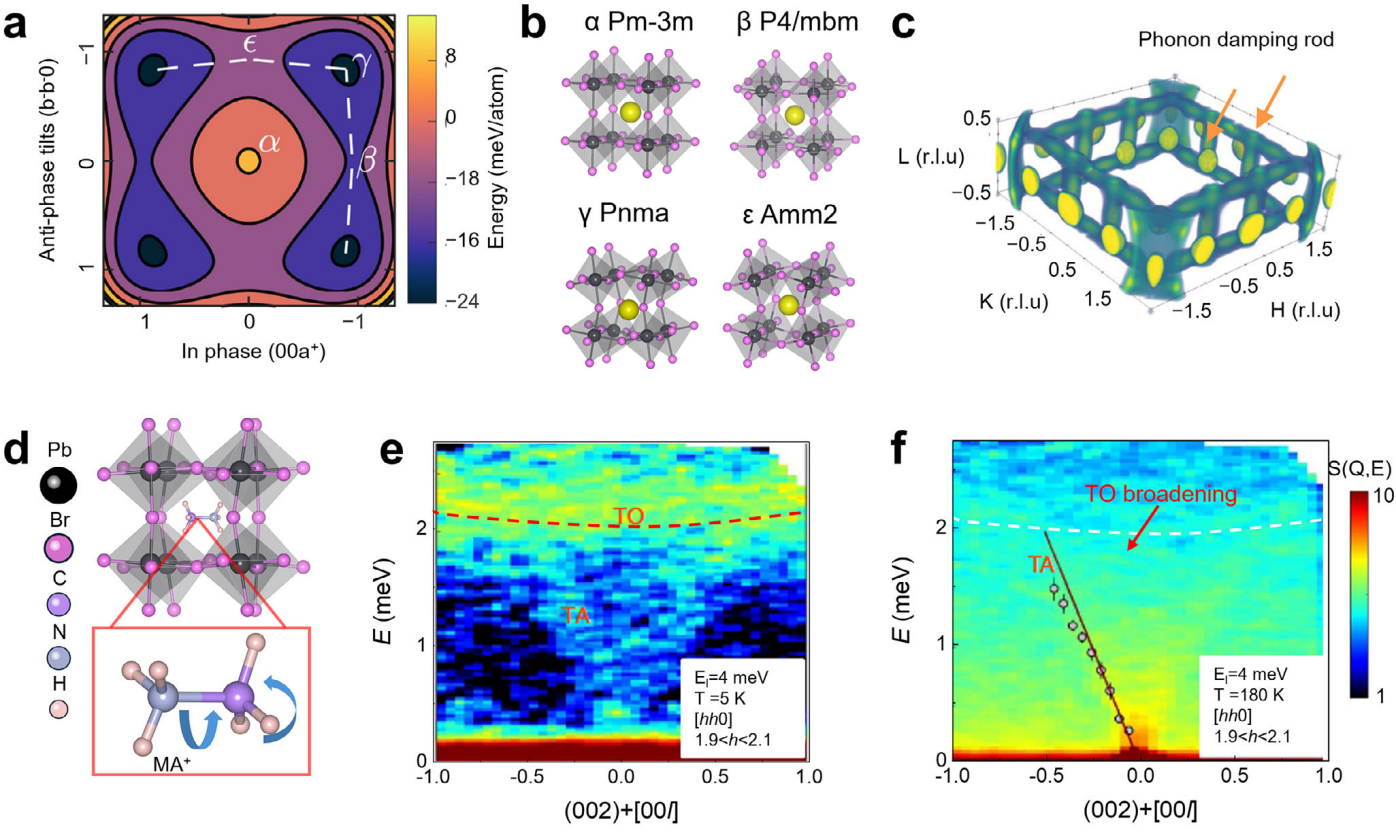
Lattice disorder in halide perovskites. (a) Octahedral tilting potential energy landscape in CsPbBr_3_. Reproduced with permission [85]. Copyright 2019, American Physical Society. (b) Tilted structures of the [PbBr] octahedra under the Pm‐3m, P4/mbm, Pnma, and Amm2 space groups, corresponding to the α, β, γ, and ε phases, respectively. At 0 K, only the orthorhombic phase is dynamically stable, whereas the cubic and tetragonal phases exist at elevated temperatures due to anharmonic vibrations that enable octahedral hopping across multiple saddle points, giving rise to a time‐averaged structure. (c) Three‑dimensional rendering of the phonon overdamping region in CsPbBr_3_. Dynamic tilting of [PbBr] octahedra results in rod‑like phonon diffuse scattering in reciprocal space, as indicated by the arrows. Reproduced with permission [101]. Copyright 2021, Nature. (d) Schematic illustration of MA^+^ cation rotation in MAPbI_3_.(e) and (f) Transverse acoustic phonons at the (220) Brillouin zone of MAPbI_3_ measured at 5 and 180 K. At 180 K, the transverse optical (TO) mode broadens due to activation of dynamic disorder of MA^+^ dipoles, leading to optical branch overdamping. Reproduced with permission [102]. Copyright 2017, Nature.

Halide perovskites exhibit pronounced defect tolerance because their soft lattices can accommodate substantial distortions while maintaining structural stability [61, 103]. A certain degree of atomic size mismatch merely distorts the lattice or alters the dimensionality of atomic clusters, as exemplified by Cs_3_Bi_2_I_6_Cl_3_ [63]. Such distortions often possess symmetry‐equivalent positions in space that share identical potential energies and thereby form double‐well potentials [104]. This feature enables halide perovskites to undergo structural hopping between potential wells under thermal activation and gives rise to dynamic structural disorder [85, 105, 106]. As shown in Figure 5a, the 2D potential energy surface of CsPbBr_3_ reveals the phase transition pathways driven by lattice thermal fluctuations. The octahedral rotations exhibit four symmetrically distributed potential energy minima. The horizontal and vertical axes correspond respectively to in‐phase rotations of the [PbBr_6_] octahedra about the c‐axis and out‐of‐phase rotations about the a‐ and b‐axes of the pseudocubic unit cell [107]. The energy extrema and saddle points correspond to distinct structural phases of CsPbBr_3_, as illustrated in Figure 5b. At low temperatures, CsPbBr_3_ adopts the orthorhombic Pnma structure with the potential energy residing near a minimum. As the temperature increases, the octahedral rotations are driven across energy barriers, and the system hops among two or four potential wells. The α, β, and ε phases manifest as averaged structures through the γ phase amid the continuous reorientation of the octahedra. Similar phase transitions and large‐amplitude dynamic fluctuations of [BX] clusters are observed in other halide perovskites such as CsSnBr_3_ and Cs_2_AgBiBr_6_ [46, 74, 108]. This behavior has been collectively termed the “crystalline liquid” character of halide perovskites [61].

Dynamic structural disorder arising from octahedral tilting and rotational fluctuations gives rise to overdamped soft modes at the Brillouin zone boundaries of halide perovskites [101, 109, 110]. Under these conditions, phonons become entirely incapable of transporting heat. As shown in Figure 5c, single‐crystal inelastic neutron scattering (INS) experiments on cubic CsPbBr_3_ have revealed a pervasive network of quasielastic diffuse scattering rods that extend continuously across the Brillouin zone boundaries. In three‐dimensional reciprocal space, these diffuse rods propagate along the <001> directions and form an interconnected network of continuous phonon damping. In real space, the phonon energies along these directions remain nearly constant at a remarkably low value of approximately 1 meV and exhibit a complete loss of propagative character [101]. A synergistic combination of computational and experimental analyses has confirmed that the atomic displacement patterns of these soft phonons at the zone boundaries correspond to collective octahedral rotations dominated by the motion of Br atoms [101]. Frozen‐phonon calculations further reveal that this rotational mode resides within a double‐well potential with a depth of approximately 26 meV/atom, an observation that unequivocally establishes the cubic phase as dynamically unstable with respect to this distortion [101]. Experimentally, these soft modes manifest as overdamped quasielastic diffuse scattering rods in the high‐temperature cubic phase. As the temperature is lowered toward the orthorhombic phase, the diffuse rods undergo a freezing transition and condense into a set of well‐defined, sharp diffraction spots [101]. This evolution from diffuse to sharp features demonstrates that the overdamped regions in the phonon dispersion of halide perovskites originate fundamentally from dynamically correlated structural fluctuations associated with octahedral rotations [101].

Another type of structural disorder in halide perovskites, distinct from that governed by the B─X lattice, originates from the organic cation moieties in organic–inorganic hybrid halide perovskites [61, 102, 111]. Organic cations such as MA^+^ and FA^+^ differ fundamentally from their spherically symmetric inorganic counterparts. They possess orientational symmetry and an intrinsic electric dipole moment. The former introduces a substantially richer spectrum of vibrational modes, including stretching and rocking of the C─N bond and rotation of the MA^+^ group beyond the simple translational rattling of the cation within the lattice cage, as illustrated in Figure 5d [112]. The latter generates picosecond dielectric noise during the disordered reorientation of the organic moiety and influences the optical lattice vibrations through long‐range Coulomb interactions [102].

Figure 5e,f compare the transverse phonon spectra of MAPbI_3_ in the orthorhombic phase at 5 K, and the tetragonal phase at 180 K. At low temperature, the transverse optical branch near 2.28 meV (TO, red dashed line) is clearly resolved and indicates an orientationally ordered state of the MA^+^ groups [102]. Upon warming to the tetragonal phase, thermal activation disrupts the hydrogen bonding that maintains the orientational order of the organic cations and unlocks the rotational degrees of freedom of the MA^+^ ions. The MA^+^ ions undergo ultrafast reorientational motion along the C─N axis with a characteristic time scale of 0.71 ps [102]. This motion induces intense scattering of the optical phonon branch. In Figure 5f, the originally well‐defined TO branch (white dashed line) becomes substantially broadened and even vanishes. The disordered nature of the MA^+^ organic cations has been corroborated by Raman spectroscopy [113], terahertz spectroscopy [114], time‐resolved optical Kerr effect measurements [115], and complementary computational studies [116].

It is worth noting that the optical phonon branches of the organic moiety in the intermediate‐frequency (25–45 THz) and high‐frequency (>80 THz) regimes contribute negligibly to thermal conductivity [112]. The transverse acoustic (TA) phonons in Figure 5e,f appear largely unperturbed. Experimental data and calculations of the vibrational contributions to thermal transport nevertheless demonstrate that the overall translational and rotational motions of the organic cations couple more strongly to the lattice than those of inorganic ions [117]. This stronger coupling accounts for the lower thermal conductivity of MAPbI_3_ (0.3 W m^−^^1^·K^−^^1^) compared to that of CsPbI_3_ (0.4 W m^−^^1^·K^−^^1^) [117].

## 5 Phonon Glass Behavior

**FIGURE 6:**
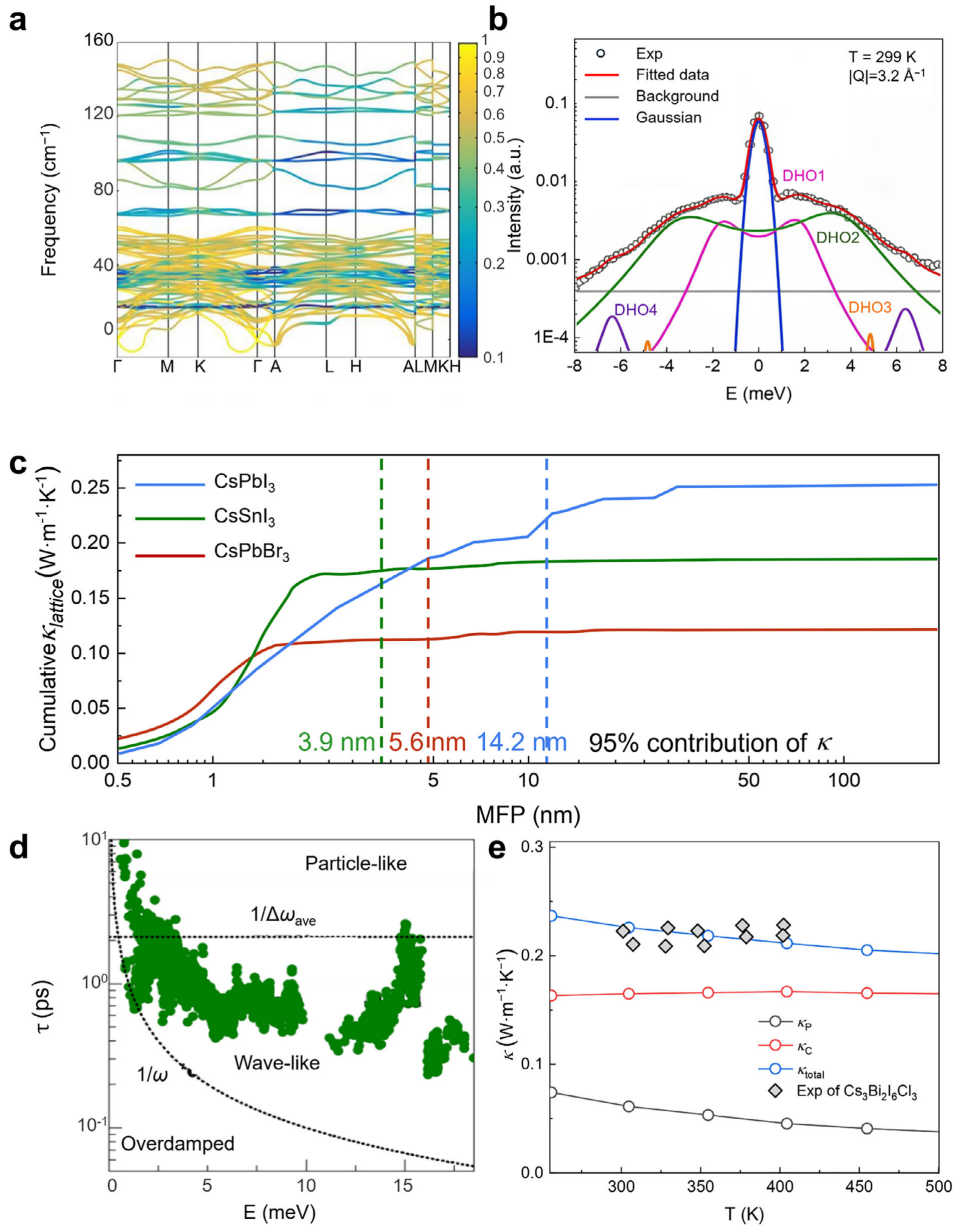
Phonon transport characteristics of halide perovskites. (a) Phonon dispersion of Cs_3_Bi_2_I_9_ calculated by density functional theory (DFT). The color scale represents the phonon participation ratio. A bluer color indicates a lower participation ratio and, consequently, a smaller contribution to thermal conductivity. Reproduced with permission [65]. Copyright 2023, Wiley‐VCH GmbH. (b) Constant‐momentum INS spectrum of CsAg_2_I_3_ at room temperature. The spectrum is fitted with a Gaussian, background, and four damped harmonic oscillator (DHO) functions labeled DHO1, DHO2, DHO3, and DHO4 in order of increasing energy. Reproduced with permission [69]. Copyright 2025, Nature. (c) Contribution of mean free path (MFP) to *κ* in CsPbBr_3_, CsSnI_3_, and CsPbI_3_. Reproduced with permission [55]. Copyright 2017, National Academy of Sciences. (d) Phonon lifetime in Cs_3_Bi_2_I_6_Cl_3_ distinguished by the Ioffe‑Regel limit (1/ω) and the Wigner limit (1/ Δω_avg_). The contribution of wave‑like coherence to thermal conductivity must be considered in halide perovskites. Reproduced with permission [100]. Copyright 2023, American Physical Society. (e) Circles denote the thermal conductivity of Cs_3_Bi_2_I_6_Cl_3_ calculated using the unified thermal transport theory. Gray diamonds represent experimental values [100].

Dynamic structural disorder not only furnishes direct experimental evidence for the strongly anharmonic lattice dynamics of halide perovskites but also provides critical microscopic clues for understanding their “phonon glass” thermal transport behavior. Calculations reveal that structural disorder in halide perovskites gives rise to strongly localized phonon modes [64, 65, 89, 118]. Figure 6a displays the phonon spectrum of Cs_3_Bi_2_I_9_. The dense optical phonon branches below 40 cm^−1^ (4.96 meV) originate from localized vibrations of Cs and I atoms [65]. These branches form a large energy gap with respect to higher‐energy phonons and constitute a dense low‐energy phonon band. Notably, the blue optical branch near 15 cm^−1^ (1.58 meV) exhibits an extremely low phonon participation ratio and a group velocity approaching zero. This low‐lying optical branch couples strongly with acoustic phonons and undergoes an avoided crossing at the Brillouin zone edge. This avoided crossing imposes a low cutoff frequency of approximately 18 cm^−1^ (2.21 meV) and results in the softening of the acoustic branch into a flattened dispersion with reduced group velocity [65].

Phonon spectra of halide perovskites obtained from DFT often contain multiple branches with imaginary frequencies. This artifact arises because the crystal structures that exhibit thermally disordered fluctuations at elevated temperatures are dynamically unstable at 0 K. Eliminating these imaginary frequencies to obtain physically meaningful phonon dispersions typically requires either freezing specific phonon modes to stabilize the structure or employing effective potentials that incorporate finite‐temperature effects [63, 70, 101].

The imaginary frequency branches that emerge within the harmonic approximation can be attributed to low‐energy overdamped soft modes induced by strong acoustic‐optical phonon scattering in halide perovskites. Experimentally, such heavily damped lattice vibrational modes can be quantitatively characterized through constant‐momentum spectra obtained from inelastic neutron scattering by fitting with damped harmonic oscillator functions. Taking the room‐temperature data of CsAg_2_I_3_ presented in Figure 6b as an example, the phonon spectrum exhibits two extensively broadened damped harmonic oscillator peaks (DHO1 and DHO2) with center frequencies both below 4 meV [69]. These peaks encompass the acoustic and low‐energy optical branches that participate in thermal transport. Notably, the full widths at half maximum of these modes substantially exceed their respective center frequencies. The spectral profile consequently evolves into broad featureless humps devoid of distinct peak positions. This characteristic indicates that the low‐energy phonons responsible for thermal transport have lost the well‐defined particle‐like propagative behavior typical of conventional crystals [69]. They instead exhibit a “phonon glass” state of thermal conduction characterized by the coexistence of propagons and diffusons akin to that observed in amorphous materials.

Within the conventional phonon gas model, the validity of phonons as propagating quasiparticles hinges on whether their MFP exceeds the phonon wavelength or the interatomic spacing [9]. The Ioffe‐Regel limit is widely regarded as the theoretical threshold beyond which this quasiparticle picture breaks down [47]. In halide perovskites, strong intrinsic phonon scattering compresses the MFP to the nanometer scale [55]. As shown in Figure 6c, 95% of the thermal conductivity in CsPbI_3_, CsPbBr_3,_ and CsSnI_3_ is contributed by phonons with MFP of merely 3–10 nm [55]. This length scale corresponds to only two to three times the unit cell parameter and indicates that conventional grain boundary scattering is no longer the dominant source of thermal resistance. Experimental estimates derived from thermal conductivity, sound velocity, and heat capacity yield an average phonon mean free path of approximately 4.3 nm for CsPbBr_3_ and the MAPbX_3_ family of perovskites [50]. The variations in thermal conductivity among these materials are therefore governed primarily by differences in group velocity and heat capacity rather than by further reductions in the MFP.

The MPF in the CsPbBr_3_ systems remains several times the interatomic spacing and has yet to strictly breach the Ioffe‐Regel limit. The extreme scenario that genuinely approaches this threshold emerges in halide perovskite derivative systems possessing more complex crystal structures. Taking Cs_3_Bi_2_I_6_Cl_3_ as an example, the experimentally estimated *l* is approximately 0.67 nm and lies on the same order of magnitude as the interatomic spacing [47]. This ultimate compression signifies that particle‐like phonon propagation has been entirely supplanted by wave‐like diffusive transport [100]. To quantitatively distinguish the contributions of these two types of heat carriers, the thermal transport modes can be classified into particle‐like phonons and wave‐like phonons based on the frequency dependence of the phonon lifetime, as illustrated in Figure 6d. Most phonon modes exhibit lifetimes concentrated within an ultrashort window of 1–10 ps and reside between the Ioffe‐Regel limit (τ = 1/*ω*) and the Wigner limit (τ = 1/Δ*ω*_avg_) [100]. These modes contribute to wave‐like tunneling (coherent) thermal conductivity (*κ*_c_). Only phonons with lifetimes exceeding the Wigner limit contribute to particle‐like thermal transport (*κ*_p_). The unified thermal transport theory successfully explains and quantifies the anomalous amorphous‐like thermal conductivity of Cs_3_Bi_2_I_6_Cl_3_ [100, 119]. Coherent phonon‐mediated thermal transport, which dominates in disordered glasses, accounts for 76% of the total thermal conductivity in Cs_3_Bi_2_I_6_Cl_3_ (Figure 6e) [100].

## 6 Summary and Outlook

We have reviewed the research progress on halide perovskites as an emerging class of low‐thermal‐conductivity thermal insulation materials. This material system has attracted widespread attention owing to its rich chemical tunability and distinctive lattice dynamics. Halide perovskites exhibit thermal transport characteristics that include weak temperature dependence of thermal conductivity, a boson‐like peak in low‐temperature heat capacity, and anomalously low sound velocities. The single‐crystalline compound Cs_3_Bi_2_I_6_Cl_3_ in particular displays anomalous amorphous‐like thermal transport and provides compelling evidence for the effective modulation of thermal conduction through structural engineering. These macroscopic thermal signatures collectively point to the inherent softness of the lattice framework and the pronounced vibrational anharmonicity of halide perovskites.

Elucidating the microscopic origins of the ultralow thermal conductivity in halide perovskites and achieving effective control over their thermal transport properties require a thorough understanding of their crystal structures, chemical bonding characteristics, and lattice dynamical processes. First, the A‐site cations undergo large‐amplitude localized rattling within the loosely bound framework of [BX_6_] octahedra and thereby serve as potent intrinsic phonon scattering centers. Second, the distinctive MVB of the B‐X linkage markedly softens the lattice and renders it highly susceptible to stretching and bending deformations. This soft lattice character endows halide perovskites with pronounced defect tolerance. Lattice distortions induced by ionic size mismatch create shallow double‐well potentials and give rise to a dynamically disordered lattice state under thermal activation. This dynamic disorder manifests as a pervasive network of overdamped phonon modes at the Brillouin zone boundaries. Concurrently, organic A‐site cations scatter low‐energy optical phonons intensely through their rotational degrees of freedom and dipole moment fluctuations. These optical modes couple strongly with the acoustic branches and further obstruct thermal transport pathways. The aforementioned multiple disorder effects collectively reflect the strong anharmonicity of the lattice vibrations. Coherent wave‐like phonons consequently supplant propagating phonons as the dominant heat carriers and ultimately give rise to the “phonon glass” state of thermal conduction characteristic of halide perovskites.

The recent advances in understanding the low thermal conductivity of halide perovskites underscore the critical importance of the intrinsic correlation between structure and lattice dynamics for achieving precise control over their thermal transport properties. Several design strategies have effectively exploited this structure‐property relationship to tune thermal conductivity. For example, varying the size of the A‐site organic group can adjust the configuration of the [BX] cage, decoupling the thermal conductivity contributions from organic chains and the inorganic framework, thereby enabling phonon engineering [57, 120, 121]. Doping or substitution at the B‐site directly alters the B─X coordination environment, reshaping thermal transport pathways by inducing polyhedral distortion and tilting [108, 110, 122]. However, systematically exploring the vast chemical space to discover low‑thermal‑conductivity halide perovskites with novel structural and compositional combinations cannot rely solely on empirical structure‑tuning approaches. The combination of high‑throughput computations and machine learning is increasingly becoming a powerful tool to accelerate this process [69, 71]. Several physical parameters highly correlated with low thermal conductivity can serve as key screening descriptors, including: (i) Debye temperature (*θ*_D_), which comprehensively reflects lattice stiffness; (ii) Grüneisen parameter (γ), as a measure of lattice anharmonicity; (iii) boson peak characteristic temperature, indicating the frequency of localized optical phonon modes; and (iv) number of atoms per unit cell (*N*), representing lattice complexity. The tunability of thermal conductivity in halide perovskites provides a versatile platform for the rational optimization of thermal insulation performance through intrinsic phonon engineering.

Based on the regulatory strategies of the structure‐performance relationship mentioned above, the ultralow thermal conductivity achieved by halide perovskites has shown broad application prospects in multiple technical scenarios. First, in the field of precision low‑temperature thermal insulation, halide perovskites, as dense solids, intrinsically offer extremely low thermal conductivity without relying on porous structures, thereby enabling efficient thermal insulation while maintaining excellent mechanical stability. This makes them suitable for demanding applications such as infrared detectors and gravitational wave detectors that require high‐precision and low‐temperature operation [123, 124]. Second, in thermoelectric energy conversion, ultralow lattice thermal conductivity is a key prerequisite for achieving a high figure of merit (*ZT*). The rich chemical tunability of halide perovskites allows the simultaneous optimization of electrical conductivity and the Seebeck coefficient, positioning them as promising emerging thermoelectric materials [125]. Furthermore, in perovskite solar cells, the inherently low thermal conductivity of the active layer directly affects the device's heat dissipation capability, potentially inducing localized hot spots and exacerbating thermally driven degradation [58]. Therefore, a deep understanding of thermal transport mechanisms and precise control of thermal conductivity are of practical significance for improving the long‑term operational stability of photovoltaic devices.

Although ultralow thermal conductivity has been widely reported for halide perovskites, several critical issues remain to be systematically investigated. These include the anomalous thermal transport behavior observed in CsPbBr_3_ [126], the ongoing controversy over the “cage” model between A‐site cations and the [BX_6_] framework [127], and the significant differences in thermal properties among bulk, thin‐film, and nanostructured forms [128]. Furthermore, the bottleneck effect induced by phase transitions and moisture‑induced degradation severely limits the long‑term environmental stability of halide perovskites as thermal insulation protective layers, hindering their practical applications [129]. In the field of thermoelectrics, the theoretically predicted performance of halide perovskites is impressive, yet the experimentally reported values fall far short of expectations [46]. In particular, improving electrical conductivity remains challenging and represents a key bottleneck for achieving high‑efficiency thermoelectric conversion [125]. Future research should integrate in situ analysis of structural phase transitions, microscopic phonon measurements, and accurate anharmonic lattice dynamics calculations to clarify the lattice dynamics mechanisms and achieve targeted control over specific phonon modes. At the same time, leveraging machine learning techniques to explore composition engineering and device integration strategies that combine air stability with high electrical conductivity will be a critical pathway toward overcoming practical application bottlenecks.

## Author Contributions

**Haolin Ye**: writing – original draft, formal analysis, visualization, data curation, conceptualization. **Bangzhi Ge**: validation, formal analysis. **Chongjian Zhou**: conceptualization, funding acquisition, writing – review and editing, project administration, supervision, formal analysis. **Yuan Yu**: conceptualization, writing – review and editing, writing – original draft, formal analysis, supervision, project administration.

## Conflicts of Interest

The authors declare no conflicts of interest.

## Data Availability

The data that support the findings of this study are available from the corresponding author upon reasonable request.
